# Community-Acquired MRSA Pyomyositis: Case Report and Review of the Literature

**DOI:** 10.1155/2011/970848

**Published:** 2011-03-02

**Authors:** Douglas P. Olson, Sarita Soares, Sandhya V. Kanade

**Affiliations:** ^1^Yale University School of Medicine, Waterbury Hospital Campus, 64 Robbins Street, Pomeroy 3, Waterbury, CT 06721, USA; ^2^Yale New Haven Hospital, New Haven, CT 06511, USA

## Abstract

Community-acquired methicillin-resistant *Staphylococcus aureus* (CA-MRSA) is responsible for a broad range of infections. We report the case of a 46-year-old gentleman with a history of untreated, uncomplicated Hepatitis C who presented with a 2-month history of back pain and was found to have abscesses in his psoas and right paraspinal muscles with subsequent lumbar spine osteomyelitis. Despite drainage and appropriate antibiotic management the patient's clinical condition deteriorated and he developed new upper extremity weakness and sensory deficits on physical exam. Repeat imaging showed new, severe compression of the spinal cord and cauda equina from C1 to the sacrum by a spinal epidural abscess. After surgical intervention and continued medical therapy, the patient recovered completely. This case illustrates a case of CA-MRSA pyomyositis that progressed to lumbar osteomyelitis and a spinal epidural abscess extending the entire length of the spinal canal.

## 1. Introduction

The differential diagnosis of fever and back pain in an intravenous drug abuser is broad. Pyomyositis is an intramuscular abscess of the large skeletal muscle groups with most initial cases in the tropics but with increasing worldwide distribution in nontropical areas as well. It is often not diagnosed until the later stages of infection [1]. Diagnosis is based on a combination of clinical, laboratory, and radiologic findings [1, 2]. Once treatment is begun, outcome generally correlates with the extent of disease. We present a case of primary pyomyositis with osteomyelitis that, despite appropriate antibiotics and drainage, progressed and extended into the epidural space, compressing the entire spinal column. The objectives of this paper are to: (1) present a case of progressive pyomyositis and (2) review the diagnosis and treatment of an infection that has been increasing in incidence in tropical and temperate climates. 

## 2. Case

A 46-year-old gentleman presented to the hospital complaining of right paraspinal back pain of 2-month duration with increasing intensity. In the days prior to admission, he had also noticed fevers, decreased appetite and overall malaise. 

The patient had a medical history of uncomplicated, untreated genotype 1 hepatitis C virus (HCV) and dyspepsia. Surgical history was unremarkable. He was not taking any medications. He lived at home with his mother. His last reported IV drug use was two months prior. He continued to smoke a pack of cigarettes a day and denied alcohol use. He was heterosexual and denied unsafe sexual practices. There was no recent travel and no history of trauma. He reported an allergic reaction to penicillin.

Initial physical examination revealed a temperature of 102.2 degrees Farenheit, a heart rate of 137 beats per minute, a blood pressure of 109/71 mmHg, and a respiratory rate of 16 breaths per minute with an oxygen saturation of 97% on room air. The patient was in moderate distress and appeared ill. His cardiovascular exam revealed a regular tachycardia, without murmurs, rubs or gallops. His lungs were clear and his abdominal exam was unremarkable. His back exam revealed spinal tenderness from L2 through L4 without fluctuance or costovertebral angle tenderness. His extremity exam was significant for a right-sided psoas sign, otherwise, strength and range of motion was intact. Neurologically there were no cranial nerves deficits, reflexes were symmetrical and normal, and distal sensation was intact. Dermatologic exam was unremarkable. 

**Figure 1:**
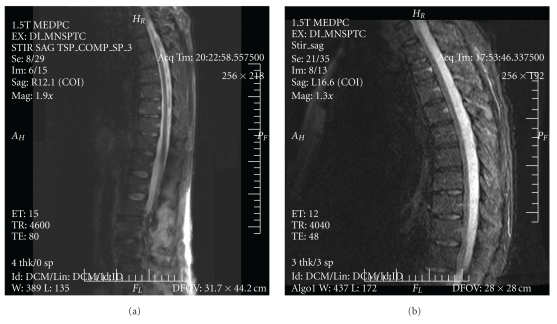
Saggittal thoracolumbar spine MRI with STIR (short T1/tau inversion recovery): day 1 (a) and day 11 (b). Serial MRI images of the thoracic and sacral spinal cord from hospitalization day 1 (a) and day 11 (b) using STIR (short T1/tau inversion recovery) sequences. Panel A shows a 12 × 3 cm multiloculated abscess of the right paraspinal musculature in the L2–L5 region, and subtle enhancement of the L3-L4 transverse processes, consistent with osteomyelitis. There is no epidural abscess. The MRI in (b) shows extensive rim enhancement of the epidural column and severe anterior compression of the spinal cord.

**Figure 2:**
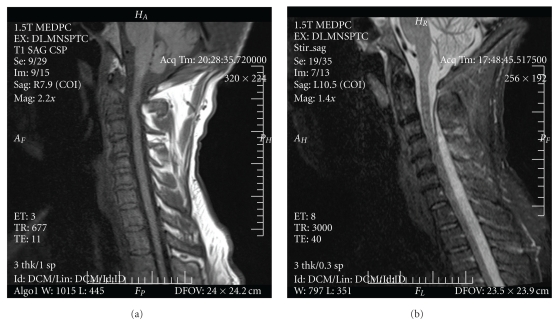
Saggittal T1 cervical spine MRI: day 1 (a) and day 11 (b). Serial MRI images of the cervical spine from hospitalization day 1 (a) and day 11 (b). (a) shows a relatively normal cervical spine with only mild degenerative disc protrusion and mild canal stenosis at C6-C7. (b) shows epidural enhancement and marked anterior compression of the spinal cord beginning at the level of C1 and continuing caudally.

Laboratory data revealed a white blood cell count of 11.2 × 10^3^ cells/mm, a hemoglobin of 11.2 gm/dL, and platelets of 220 × 10^9^/L. Sodium was 131 mEq/L and the remainder of his chemistry panel, coagulation studies and liver function tests were normal. There was no anion gap. Erythrocyte sedimentation rate was 80 mm/hr. An initial chest X-ray was normal. Blood and urine cultures were drawn and the patient was started on vancomycin. A magnetic resonance image (MRI) with gadolinium contrast of the lumbar and thoracic spine done on the day of admission revealed a large multiloculated abscess measuring 12 cm craniocaudally by 3 cm axially within the right posterior paraspinal musculature from L2 through L5, with subtle enhancement of the L3 and L4 right transverse processes consistent with osteomyelitis. There was dural enhancement at L3 and L4 but no epidural abscess (Figure 1(a)), and his cervical spine was unremarkable (Figure 2(a)). A pigtail catheter drain was inserted into the paraspinal abscess on hospital day 2. Blood and abscess cultures grew methicillin-resistant *Stapyolococcus aureus *(MRSA) sensitive to vancomycin with a minimum inhibitory concentration [MIC] of ≤0.5 *μ*g/mL.

The patient improved clinically by hospital day 5. White blood cell count, which initially had increased, trended down to normal. His catheter was not draining any fluid. The peak vancomycin level measured on day three was 32 mcg/mL, with a trough of 12 mcg/mL. A transesophageal echocardiogram ordered on admission for initial concern for endocarditis revealed a small filamentous echodensity in the right anterior mitral valve leaflet consistent with a small ruptured chordae without evidence of vegetations of valvular regurgitation. A human immunodeficiency virus (HIV) antibody test was negative. His HCV viral load was 1.32 × 10^6^, with a normal alpha-fetoprotein level and an ultrasound consistent with cirrhosis but without focal masses. Surveillance cultures remained with no growth. The patient inadvertently removed the pigtail catheter 4 days after insertion, on hospital day 6. He remained afebrile. 

On hospital day 11, the patient complained of new onset tingling in his fingertips bilaterally with upper extremity weakness and minimal discomfort of his shoulders and chest muscles. Exam at this time showed no meningismus, normal cranial nerve function and reflexes. Strength was decreased bilaterally and symmetrically on shoulder abduction (2/5), elbow flexion and extension (4/5), abductor pollicis brevis function (3/5), finger abduction (4/5) and finger extension (3/5). There was continued, decreased right paraspinal tenderness but an improved psoas sign, with no new findings otherwise. A repeat MRI performed at this time revealed an extensive rim-enhancing epidural collection posterior to the spinal cord and cauda equina that extended from the level of the first cervical vertebra to the sacrum, with continuous and severe cord compression against the anterior spinal column (Figures 1(b) and 2(b)) consistent with a large spinal epidural abscess.

The patient was emergently taken to the operating room on day 11 for decompression and epidural abscess debridement. Cultures taken from the epidural abscess intraoperatively grew MRSA, and the MIC to vancomycin remained <0.5 ug/mL in this culture, which excluded both a vancomycin intermediate resistant organism (where the defined MIC is between 4–8 ug/mL) and a fully vancomycin resistant organism (where the MIC is ≥16 ug/mL). He had no neurological impairment and was discharged on hospital day 18 to a short-term rehabilitation center for physical and occupational therapy and a six-week course of intravenous vancomycin, chosen based on microbiological culture data, where he recovered without sequelae. 

## 3. Discussion

Pyomyositis is a suppurative infection of the large skeletal muscles without an apparent spread from contiguous structures. Initially reported largely in the tropics, it has become a disease of worldwide occurrence, with many cases reported in temperate climates [1]. Thought to be due to seeding of a muscle from transient bacteremia, the exact pathophysiology is unknown, as only 5% to 37% of patients with pyomyositis are bacteremic [1]. *Staphylococcus aureus* is responsible for greater than 70% of infections [2], though multiple other organisms have been reported [3]. Cases of MRSA pyomyositis have been increasing reported to date [4]. Comorbid conditions may contribute to the pathogenesis of pyomyositis, with approximately half of infected patients having diabetes mellitus, cirrhosis, aplastic anemia, sickle cell disease, rheumatologic disease, malignancy, a history of intravenous drug use or being immunosuppressed either by the use of a medication or by HIV [5, 6]. 

The most commonly involved muscles are those in the thigh and gluteal region, though infection of the deltoid, psoas, biceps, gastrocnemius, and paraspinal region have also been reported [6]. Classically, pyomyositis is described as occurring in three stages [1]. Stage 1 manifests as crampy muscle pain and low grade fever. There is often no sign of underlying muscle or soft tissue swelling or infection. Patients often do not present at this stage due to the vague nature of the complaints. If they are seen by a physician, the condition is often misdiagnosed. Stage 2 begins two to three weeks after the initial onset of symptoms and includes worsening muscle pain, swelling, erythema and fever. A localized purulent collection has developed by this stage, and patients often seek medical attention. If patients are not treated in this stage, they progress to stage 3, which include sepsis and clinical signs of toxicity. Diagnosis involves recognition of the appropriate signs and symptoms as well as a high index of suspicion. 

Adjunctive laboratory testing is often not helpful, with variations in the white blood cell count, C-reactive protein and/or erythrocyte sedimentation rate being dependent on underlying diseases, especially, HIV, and often being nonspecific [7]. Despite obvious muscle destruction, serum creatine kinase and aldolase are often normal [8]. Blood cultures are positive only in a minority of patients. MRI with gadolinium contrast is the preferred diagnostic test, if available, showing low signal intensity on T1 weighted images and high signal intensity with diffuse borders and contrast enhancement of T2 weighted images [9]. It is often difficult to diagnose without MRI given the vague and subacute presentation. While other imaging modalities have been used in resource-poor settings, MRI with gadolinium contrast is the gold-standard imaging test especially in cases of infections that affect more than the extremities, as it can provide a more specific diagnosis if there are multiple infections simultaneously (i.e., pyomyositis and osteomyelitis, as in this case) [10].

Treatment of pyomyositis is dependent on the stage of the infection at diagnosis [1]. Stage 1 can be treated with oral antibiotics. More advanced stages require intravenous antibiotics with initial broad coverage for all potential pathogens, keeping in mind that patients with underlying medical comorbidities have a higher risk of gram-negative infections. Often times, interventional radiology or surgical intervention is necessary to drain abscesses and fluid collections. Complications of pyomyositis include muscle scarring, residual weakness, osteomyelitis, septic arthritis, pericarditis and septic shock, among others [1]. With appropriate therapy, patients usually recover well without sequelae. 

What is unique to the present case is that the patient's infection progressed despite appropriate antibiotic therapy and drainage. It also highlights the close anatomical relationship of the involved progressive infection: the initial pyomyositis of the paraspinal muscle, which lies just posterior to the lumbar transverse processes, and psoas muscle, which abuts the lateral portion of the vertebral body, had eroded into the lumbar vertebrae and eventually extended into the spinal canal. Despite the dramatic clinical deterioration of our patient, he made a complete recovery without any permanent neurological deficits.
